# Clinical Outcome and Molecular Profile in Patients with *DDX41* Mutation Hot-Spots

**DOI:** 10.3390/hematolrep17030026

**Published:** 2025-05-08

**Authors:** Nadia Toumeh, Yazan Jabban, Ahmad Nanaa, Rong He, David Viswanatha, Dragan Jevremovic, James M. Foran, Cecilia Y. Arana Yi, Antoine N. Saliba, Mehrdad Hefazi Torghabeh, William J. Hogan, Mithun V. Shah, Abhishek A. Mangaonkar, Mrinal M. Patnaik, Hassan B. Alkhateeb, Aref Al-Kali

**Affiliations:** 1 Internal Medicine, Mayo Clinic, Rochester, MN 55905, USA; toumeh.nadia@mayo.edu; 2 Division of Hematology, Mayo Clinic, Rochester, MN 55905, USA; jabban.yazankhaireldien@mayo.edu (Y.J.); saliba.antoine@mayo.edu (A.N.S.); hefazitorghabeh.mehrdad@mayo.edu (M.H.T.); hogan.william@mayo.edu (W.J.H.); shah.mithun@mayo.edu (M.V.S.); mangaonkar.abhishek@mayo.edu (A.A.M.); patnaik.mrinal@mayo.edu (M.M.P.); 3 John H. Stroger, Jr. Hospital of Cook County, Chicago, MN 60612, USA; ananaa2@uic.edu; 4 Division of Hematopathology, Mayo Clinic, Rochester, MN 55905, USA; he.rong@mayo.edu (R.H.); viswanatha.david@mayo.edu (D.V.); jevremovic.dragan@mayo.edu (D.J.); 5 Division of Hematology & Medical Oncology, Mayo Clinic, Jacksonville, FL 32224, USA; foran.james@mayo.edu; 6 Division of Hematology/Oncology, Mayo Clinic, Scottsdale, AZ 85054, USA; aranayi.cecilia@mayo.edu

**Keywords:** *DDX41*, MDS, AML

## Abstract

**Background/Objectives:** *DDX41*, DEAD-box RNA helicase 41 gene located on chromosome 5q25.3, is one of the most mutated genes in patients with germline predisposition to myeloid neoplasms. Germline and somatic mutations often have different locations and patterns of mutation, with some hotspots displaying diversity based on ethnicity. We aimed to explore clinical outcomes in patients with various *DDX41* hot-spot mutations. **Methods**: This was a retrospective study of patients at Mayo Clinic with *DDX41* mutation identified through Next Generation Sequencing (NGS) between 2018 and 2024. We completed unadjusted comparisons using continuous or categorical variables, and survival rates were assessed using the Kaplan–Meier method and cox regression analysis. **Results**: Overall survival appears to be higher in those with p.M1| when compared to p.Asp140GlyFs*2 and p.Arg525His, with comparable survival between p.Arg525His and p.Asp140GlyFs*2. Among males with p.M1| who underwent bone marrow transplant, those who underwent bone marrow transplant appeared to have lower survival rates, although not statistically significant. Our study was limited by a small sample size, therefore limiting our ability to reach significance. **Conclusions**: Our findings suggest potential implications for clinical outcomes based on *DDX41* mutation hot-spots.

## 1. Introduction

The DEAD-box RNA helicase 41 (*DDX41*) gene is located on chromosome 5q35.3 and is involved in several cellular functions. As part of the RNA helicase family, this gene is thought to be involved in RNA splicing and ribosome biogenesis [1,2]. *DDX41* interacts with spliceosome proteins, and defects in the gene can lead to dysfunction in alternative splicing. The gene also acts as a pattern recognition receptor that detects pathogens, therefore playing a role in innate immunity [2]. The mechanism with which mutations in the gene predispose a patient to myeloid malignancies is not completely understood; *DDX41* is one of the most commonly mutated genes in those with myeloid malignancies with germline predisposition [1,3]. Patients with *DDX41* mutation who go on to develop myeloid neoplasms often have a germline heterozygous mutation and acquire a somatic mutation in the second *DDX41* allele usually in the sixth decade of life. This “second hit” typically leads to development of myelodysplastic syndrome (MDS) or acute myeloid leukemia (AML), and there is an increased frequency of disease progression in males compared to females [2]. Up to 80% of *DDX41* germline mutation carriers who develop MDS/AML have an additional somatic mutation of the gene [4].

The World Health Organization (WHO) classification in 2016 recognized myeloid neoplasms with germline predisposition as separate clinical entities [3]. As reported by Alkhateeb et al., the most common pathogenic mutation observed in a group of 33 patients with *DDX41* was M1| [5]. In another study of 191 patients with *DDX41* mutation, other frequent *DDX41* variants were p.Arg525His and p.Asp140GlyFs*2 [6]. The hot-spot mutation p.M1| has also been found in other studies to be more common compared to other locations in those with germline mutation [7]. Our study investigates clinical outcomes and molecular profiles of patients with different *DDX41* mutation (m) hot-spots. Identifying patients with germline mutations such as *DDX41* carries implications for treatment options, stem cell transplant donor eligibility, and the need for genetic counseling [3].

## 2. Materials and Methods

We conducted a single institution retrospective study of patients at Mayo Clinic with pathogenic *DDX41* mutations, diagnosed through our next generation sequencing (NGS) between 2018 and 2024. More specifically, this was the myeloid NGS obtained either at or shortly after diagnosis. An appropriate institutional review board (IRB) approval was obtained. The data regarding mutations was recorded at the time of the patient’s first NGS that identified a *DDX41* mutation. Myeloid neoplasms were diagnosed based on the WHO 2022 diagnostic criteria [8]. We classified patients as having germline *DDX41* variants or somatic variants based on confirmed germline testing results, although only 32 patients in this study (47%) chose to undergo germline testing. Germline testing was performed using either peripheral blood, or buccal/skin samples. Our analysis primarily focused on differences between the most frequent *DDX41* hot-spot variants: p.M1|, p.Arg525His, and p.Asp140GlyFs*2Gly. An unadjusted comparison using continuous or categorical variables was completed, with survival rates assessed using the Kaplan–Meier method and cox regression analysis. The median overall survival (mOS) was calculated from the time of NGS to the last follow-up or death, utilizing BlueSky Statistics V.10.3.4. Due to the limited number of cases in some variant groups, the mOS was not reached. Therefore, survival probabilities at the 12- and 24-month points were used as an alternative measure to evaluate survival outcomes. We compared our study findings with the findings published in Makishima et al. [9].

## 3. Results

In our study, we included 68 patients who were identified to have pathogenic *DDX41* mutation on NGS. We excluded patients who had a *DDX41* mutation that was only a variant of undetermined significance (VUS). Of the 68 patients included, 30 had MDS, 23 had AML, 4 had MPN, 6 were *DDX41* carriers, and 5 carried other diagnoses: clonal cytopenia of undetermined significance (CCUS) and clonal hematopoiesis with indeterminate potential (CHIP). The majority of the patients were male (M–F 48:20) with a median age of diagnosis of 72 years (range 30–93). A total of 48 of the patients with *DDX41* pathogenic mutation had normal cytogenetics, and 20 (29%) of the patients underwent bone marrow transplantation. Table 1 includes the baseline characteristics of all 68 patients: diagnosis, median age at diagnosis, DDX41 median variant allele frequency (VAF), cytogenetics, median bone marrow blast percentage, and confirmed germline mutation (Table 1). In Table 2, we focus on patients with only p.M1|, p.Arg525His, and p.Asp140GlyFs*2, and describe the clinical characteristics of these patients as well as overall survival.

**Figure 1:**
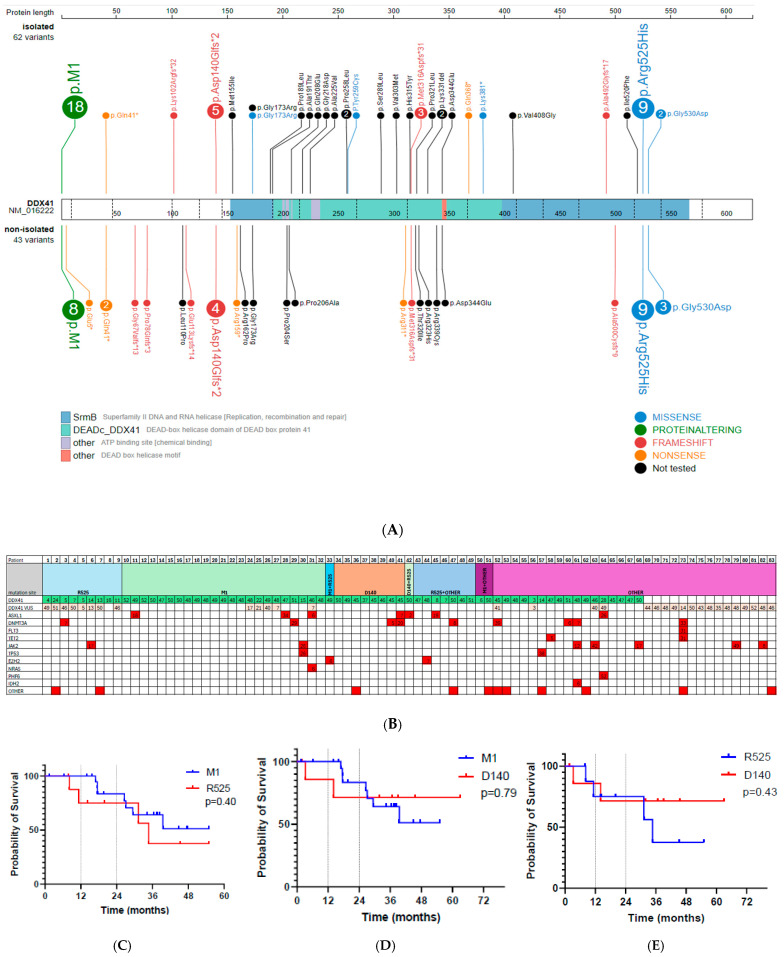
(**A**) Distribution of *DDX41* mutations, with those with isolated *DDX41* mutation above the gene box and co-mutated *DDX41* patients below the gene box. Black circles indicate patients without available genetic alteration information; (**B**) patterns of co-mutation with respective VAF among *DDX41* hot-spots, with “other” demonstrating patients with mutation other than p.M1|, p.Arg525His, p.Asp140GlyFs*2; (**C**) Kaplan–Meier survival probability in p.M1| compared to p.Arg525His; (**D**) Kaplan–Meier survival probability in p.M1| compared to p.Asp140GlyFs*2; (**E**) Kaplan–Meier survival probability in p.Arg525His compared to p.Asp140GlyFs*2.

Mutations: Mutation hot-spots included 9 patients with p.Arg525His only, 23 p.M1| only, 8 p.Asp140GlyFs*2 only, 7 p.Arg525His with other than p.M1|, and 1 patient with p.Arg525His and p.Asp140GlyFs*2 co-mutation, 1 patient had p.M1| and p.Arg525His, and 2 patients had p.M1| hot-spot with non-p.Arg525His and p.Asp140GlyFs*2. Lastly, 17 patients had mutations in locations other than specified in this study (Figure 1A).

Characteristics: The median age of diagnosis was 72 years in p.M1|, 69 years in p.Arg525His, and 63.5 years in p.Asp140GlyFs*2 mutation groups (*p* = 0.77). Patients with p.M1|, compared to p.Arg525His and p.Asp140GlyFs*2 hot-spot mutations had higher median hemoglobin level, white count level, and bone marrow blast percentage, although this was not statistically significant (*p* = 0.68, *p* = 0.24, *p* = 0.57). Patients with p.M1| were more likely, although not statistically significant, to carry normal cytogenetics, with around 90% of patients having normal cytogenetics (*p* = 0.17). This group also had the highest proportion of male patients compared to p.Arg525His and p.Asp140GlyFs*2 (*p* = 0.56). When comparing p.M1| to p.Arg525His, those with p.M1| had a higher percentage of *DDX41* variant allele frequency (VAF) percentage (48% vs. 11%, *p* = 0.001). As for germline mutations, 6 of the 23 patients with p.M1| had confirmed germline mutation, as well as 2 of the patients with only p.Asp140GlyFs*2 mutation and 1 of the patients with only p.Arg525His mutation.

Co-mutations: When looking at co-mutation prevalence, all patients had low mean number of co-mutations (p.M1| 0.3, compared to 0.6 in p.Arg525His and 0.5 in p.Asp140GlyFs*2 (*p* = 0.63)). A total of 78% of patients with p.M1| had isolated *DDX41* mutation, compared to 56% of patients with p.Arg525His and 62% of patients with p.Asp140GlyFs*2 mutation (*p* = 0.15). The most common co-mutation in p.M1| hot-spot carriers was *ASXL1,* found in 13% of patients, while the most common co-mutation in p.Asp140GlyFs*2 was *DNMT3A*, found in 29% of patients. p.Arg525His patients had various co-mutations (*JAK2*, *CBL*, *DNMT3A*, *SETBP1)*, but none were significantly more common than the others. Figure 1B displays an overview of co-mutation prevalence in the 68 patients that we studied.

Survival: We also looked at overall survival data amongst the hot-spot mutation groups, at 12 and 24 months. At 12 and 24 months, overall survival rate was 100% and 83% in the p.M1| group, compared to 75% survival rate at 12 months and 24 months for those with p.Arg525His (*p* = 0.40, Figure 1C). The p.Asp140GlyFs*2 group had 86% and 74% survival at 12 and 24 months, respectively; again a lower survival rate than those with M1| at those time periods (*p* = 0.79, Figure 1D). At 12 and 24 months, patients with p.Asp140GlyFs*2 and p.Arg525His had comparable survival probability (Figure 1E, *p* = 0.43). Among patients with only p.M1| mutation (N = 23), four of those patients underwent bone marrow transplantation (BMT). Three of those patients had high risk MDS, and one patient had AML. Those who underwent BMT = had lower median survival compared to those who did not undergo BMT (median overall survival of 63 vs. 158 months, *p* = 0.38).

## 4. Discussion

Mutations in *DDX41* account for 2–5% of patients with myeloid neoplasms, with the predominant germline mutations being frameshift, truncation, and missense [1]. The most frequent *DDX41* germline variant in a retrospective study completed by Bataller et al., in keeping with our study, was p.M1| [3]. As seen in our study, p.M1| demonstrates male predominance and higher likelihood of having normal cytogenetics [10]. Patients with p.M1| in our study were also likely to have fewer co-mutations, similar to Makishima et al. [9].

Our study also supports other findings in the multi-institutional study completed by Makishima et al. [9]. In that study, there were 346 patients with a myeloid neoplasm and *DDX41* mutation, of which 11 patients had p.M1| only, 15 had p.Arg525His only, and 24 patients had p.Asp140GlyFs*2 only. Similar to our findings, only 2 of the 346 had p.M1| and p.Arg525His co-existing hot-spots (2% in our study). Only 13 patients (3%) in Makishima et al. had co-existing p.Arg525His and p.Asp140GlyFs*2 mutation, representing similar findings to our study (2% in our study).

Patients in Makishima et al. had a low mean number of co-mutations, with p.M1| hot-spot mutation carriers having a mean number of 0.3 (range 0–2), compared to a mean of 0.9 (range 0–2) in p.Arg525His and 0.5 (range 0–3) in p.Asp140GlyFs*2 (which is similar to our study). In Makishima et al., we found that p.M1| were more likely to have isolated *DDX41*, compared to all other mutation sites (*p* = 0.01). This finding is also reported in Duployez et al., where 191 patients with *DDX41* mutation were studied and p.M1| was not significantly associated with co-mutations [6,9]. Patients in Makishima et al. with all other *DDX41* mutations had a mean co-mutation number of 1.1 (range 0–10, *p* = 0.03). The most common co-mutations in p.Asp140GlyFs*2 were *SETBP1*, *DNMT3A*, and *SRSF2*. In p.Arg525His, the most common co-mutation was *ASXL1*. The patients with only p.M1| hot-spot had a variety of co-mutations including *ASXL1*, *DNMT3A*, and *SETBP1* [9].

As for survival probability, those with p.M1| at 12 and 24 months had higher survival rates in our study, compared to p.Asp140GlyFs*2 and p.Arg525His, although not statistically significant. At 12 and 24 months, patients with p.Asp140GlyFs*2 and p.Arg525His had comparable survival probability. We also found that those with p.M1| and who underwent bone marrow transplant had lower median survival, although again not statistically significant.

Our study has several implications when it comes to taking care of patients found to have *DDX41* mutation. We believe that there is clinical utility in delineating certain hot-spot mutations present in those with *DDX41* mutation. Although our study was limited by sample size, we believe that certain hot-spot mutations may affect the patient’s success with bone marrow transplant and their likelihood of carrying other mutations known to play a role in myeloid neoplasm. For example, recent studies have noted “observation” as an option in patients who were enriched with p.Arg525His [11]. We hope to continue to expand on this data, allowing us to draw more significant conclusions.

### Study Limitations and Future Directions

We would like to mention some of the limitations of our study. First of all, our sample size was small, reflecting the need for emerging studies of the *DDX41* mutation. In addition, the retrospective nature is also inferior to that of a prospective study that analyzes patients with *DDX41* mutation. Additionally, not all of the patients in our sample underwent germline testing, therefore limiting some of the conclusions we can draw regarding the population.

## 5. Conclusions

The p.M1| hot-spot in the gene was significantly associated with higher VAF percentage and is more commonly seen in germline *DDX41* mutation. Co-mutation frequency differed by hot-spot mutation while p.Arg525His as a second mutation was seen mainly in cases other than p.M1|/pAsp140GlyFs*2. Overall survival appears to be higher in those with p.M1| compared to p.Arg525His and p.Asp140GlyFs*2, though small sample size limits our ability to draw significant conclusions. Additionally, those with M1| only mutation who underwent p.M1|bone marrow transplant had lower survival, although not statistically significant. Our findings suggest potential implications for clinical outcomes based on *DDX41* mutation hot-spots.

## Figures and Tables

**Table 1:** General characteristics of 68 patients with *DDX41* mutation identified on NGS (next generation sequencing). N: number; MDS: myelodysplastic syndrome; AML; acute myeloid leukemia; MPN: myeloproliferative neoplasm; CCUS; clonal cytopenia of undetermined significance; CHIP: clonal hematopoiesis of indeterminate potential; VAF: variant allele frequency; BM: bone marrow.

Variable	Patients Identified to Have *DDX41* Mutation (N = 68)
Median age, yrs (range)	72 (30–93)
Male sex, N (%)	48 (70%)
Diagnosis	
MDS, N (%)	30 (44%)
AML, N (%)	23 (34%)
MPN, N (%)	4 (6%)
DDX41 carrier, N(%)	6 (8%)
CCUS, N (%)	4 (6%)
CHIP, N (%)	1 (1%)
Abnormal cytogenetics, N (%)	20 (29%)
Median *DDX41* VAF %	15%
AML progression, N (%)	8 (16%)
Underwent BM transplantation	20 (29%)
Confirmed germline mutation	16 (24%)
Median BM blast%	10%

**Table 2:** Characteristics and hematological features of mutated *DDX41*, with comparison between hot-spots p.M1|, p.Arg525His, and p.Asp140GlyFs*2. Cohort 1: p.M1|, Cohort 2: p.Arg525His, Cohort 3: p.Asp140GlyFs*2. N: number; MDS: myelodysplastic syndrome; AML; acute myeloid leukemia; MPN: myeloproliferative neoplasm; CCUS; clonal cytopenia of undetermined significance; VAF: variant allele frequency; BM: bone marrow.

Variable	*DDX41* p.M1|, N = 23	*DDX41* p.Arg525His, N = 9	*DDX41* p.Asp140GlyFs*2, N = 8	*p* Value
**Median age, yrs (range)**	72 (30–83)	69 (59–90)	63.5 (60–82)	0.77
**Male sex, N (%)**	15 (65%)	7 (78%)	7 (88%)	0.56
**Diagnosis**				
MDS, N (%)	9 (39%)	5 (56%)	7 (88%)	
AML, N (%)	6 (26%)	2 (22%)	1 (12.5%)	
MPN, N (%)	1 (4%)	1 (11%)	0 (0%)	
*DDX41* carrier, N(%)	3 (13%)	0 (0%)	0 (0%)	
CCUS, N (%)	3 (13%)	0 (0%)	0 (0%)	
**Mean number of co-mutations, (range)**	0.3 (0–2)	0.6 (0–2)	0.5 (0–2)	0.63
**Co-mutations**				
*ASXL1*, N (%)	3 (13%)	0 (0%)	1 (13%)	0.79
*JAK2*, N (%)	1 (4%)	1 (11%)	0 (0%)	0.69
*DNMT3A*, N (%)	1 (4%)	1 (11.1%)	2 (29%)	0.27
*TP53*, N (%)	1 (4%)	0 (0%)	0 (0%)	1.00
*NRAS*, N (%)	1 (4%)	0 (0%)	0 (0%)	0.59
**Abnormal cytogenetics, N (%)**	2 (9%)	3 (33%)	2 (25%)	0.17
**Median *DDX41* VAF % (range)**	48 (15–52)	11 (4–24)	45 (37–50)	<0.001
**AML progression, N (%)**	1 (5.9%)	2 (25%)	0 (0%)	0.28
**Labs**				
Median hemoglobin (g/dL)	11.2	10.2	10.4	0.68
Median platelet (×10^9^/L)	94	96	153	0.64
Median white blood count (×10^9^/L)	2.8	2.5	2.3	0.24
Median BM blast %	12	11	7	0.57
**Overall survival (OS)**				
12-month OS (%)	100%	75%	86%	Cohort 1 vs. 2: 0.41 Cohort 1 vs. 3: 0.79 Cohort 2 vs. 3: 0.43
24-month OS (%)	83%	75%	71%	

## Data Availability

The original contributions presented in this study are included in the article. Further inquiries can be directed to the corresponding author.

## Abbreviations

The following abbreviations are used in this manuscript:

NGS	Next Generation Sequencing
BMT	Bone marrow transplant
CHIP	Clonal hematopoiesis of indeterminate potential
OS	Overall survival
MDS	Myelodysplastic syndrome
AML	Acute Myeloid leukemia
MPN	Myeloproliferative neoplasm
CCUS	Clonal cytopenia of undetermined significance
